# Multiplanar and two-dimensional imaging of central airway stenting with multidetector computed tomography

**DOI:** 10.1186/2049-6958-7-27

**Published:** 2012-08-31

**Authors:** Mehmet Akif Ozgul, Guler Ozgul, Erdogan Cetinkaya, Yasin Abul, Gamze Kirkil, Ekrem Cengiz Seyhan, Emine Kamiloglu, Sule Gul

**Affiliations:** 1 Yedikule Chest Diseases and Chest Surgery, Education and Research Hospital, Istanbul, Turkey; 2 Faculty of Medicine, Department of Pulmonary Medicine, Karadeniz Technical University, Trabzon, Turkey; 3 Faculty of Medicine, Department of Pulmonary Medicine, Firat University, Elazig, Turkey

**Keywords:** Airway obstruction, Endobronchial stent, Multidetector computed tomography (MDCT)

## Abstract

**Background:**

Multidetector computed tomography (MDCT) provides guidance for primary screening of the central airways. The aim of our study was assessing the contribution of multidetector computed tomography- two dimensional reconstruction in the management of patients with tracheobronchial stenosis prior to the procedure and during a short follow up period of 3 months after the endobronchial treatment.

**Methods:**

This is a retrospective study with data collected from an electronic database and from the medical records. Patients evaluated with MDCT and who had undergone a stenting procedure were included. A Philips RSGDT 07605 model MDCT was used, and slice thickness, 3 mm; overlap, 1.5 mm; matrix, 512x512; mass, 90 and kV, 120 were evaluated. The diameters of the airways 10 mm proximal and 10 mm distal to the obstruction were measured and the stent diameter (D) was determined from the average between D upper and D lower.

**Results:**

Fifty-six patients, 14 (25%) women and 42 (75%) men, mean age 55.3 ± 13.2 years (range: 16-79 years), were assessed by MDCT and then treated with placement of an endobronchial stent. A computed tomography review was made with 6 detector Philips RSGDT 07605 multidetector computed tomography device. Endobronchial therapy was provided for the patients with endoluminal lesions. Stents were placed into the area of stenosis in patients with external compression after dilatation and debulking procedures had been carried out. In one patient the migration of a stent was detected during the follow up period by using MDCT.

**Conclusions:**

MDCT helps to define stent size, length and type in patients who are suitable for endobronchial stinting. This is a non-invasive, reliable method that helps decisions about optimal stent size and position, thus reducing complications.

## Background

Computed tomography is an important diagnostic tool in lung cancer management. Particularly multidetector computed tomography (MDCT) provides guidance for primary screening of the central airways. The system is capable of providing high-resolution images of the central airways in only a few seconds. MDCT offers faster approach, greater coverage of the image, and improved spatial resolution compared to single-detector helical computerized tomography imaging [1-3]. Besides providing anatomic imaging of tracheobronchial tree, the use of dynamic expiratory MDCT imaging can also provide functional information about the airways. A number of endobronchial procedures as well as laser photocoagulation, brachytherapy and endobronchial stenting can be performed with the guidance of MDCT. This technique can also provide information about the patency of the airways distal to an obstruction [4]. Preoperative airway evaluation is very important in patients who are candidates for endobronchial treatment. MDCT can also be reconstructed as a two or three dimensional image to help to overcome the limitations of axial images by providing a more anatomically meaningful display of complex airways stenoses [5,6].

## Methods

The present investigation conforms to the principles outlined in the Declaration of Helsinki. The study was approved by the local ethics committee. Our study design was retrospective and data were collected from an electronic database and from the medical records. Diagnosis of airway obstruction was confirmed by symptoms, physical examination and radiological findings. Patients considered unsuitable for surgical resection were included in the study. 56 patients who had been evaluated with MDCT and who had undergone a stenting procedure following endobronchial treatment were included in the study. Stents were implanted at our interventional bronchoscopy unit under general anesthesia using rigid bronchoscopy as well as flexible bronchoscopy as necessary. Other endobronchial procedures including balloon dilatation, mechanical debulking, laser photo resection or electro surgery were used alone or in combination as necessary. Patients, 14 females (25%) and 42 males (75%), were aged between 16 and 79 years (mean 55.3 ± 13.2 years); 37 patients (66%) were diagnosed with malignancy and the remaining 19 (34%) had benign diseases. The diagnoses of airway obstruction and the locations of stenosis that required stent placements are summarized in Tables 1 and 2.

**Table 1:** Causes of airway obstruction and distribution of patients

**Causes of airway obstruction**	**Number of patients (%) N = 56**
Lung cancer	34 (60.7%)
Post-intubation stenosis	18 (32.1%)
Thyroid cancer	1 (1.8%)
Esophageal cancer	2 (3.6%)
Intrathoracic goitre	1 (1.8%)

**Table 2:** Location of airway obstruction and distribution by patient

**Endobronchial stenosis**	**Number of patients (%) N = 56**
Tracheal	29 (51.8%)
Tracheobronchial	13 (23.2%)
Bronchial	14 (25%)

**Figure 1:**
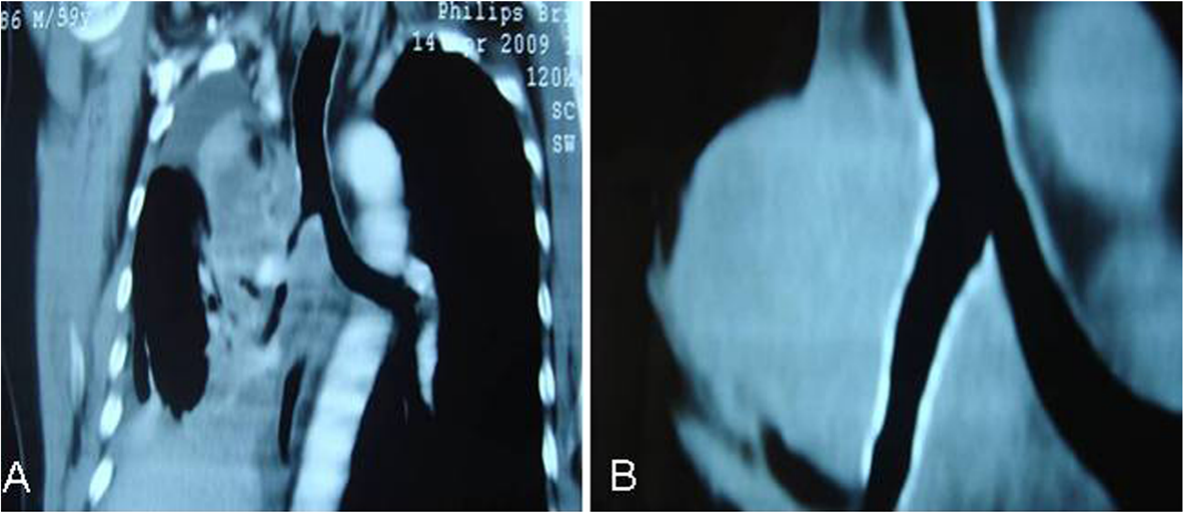
**A Follow up MDCT of a patient diagnosed as a squamous cell lung cancer.B** MDCT of a patient with alveolus stent in the right main and the intermediate bronchi. A 59 year old male patient complained of shortness of breath and back pain, was diagnosed as a squamous cell lung cancer, received chemotherapy and radiotherapy treatment. Follow up MDCT revealed the appearance of an extrinsic obstruction in the right main and the intermediate bronchi.

A Philips RSGDT 07605 model MDCT with 6 detectors was used for the thoracic MDCT examination of all patients. Parameters used for the examination included: slice thickness, 3 mm; overlap, 1.5 mm; matrix, 512 × 512; mass, 90 and kV, 120. Two dimensional reconstruction was applied following suitable angling and rearrangement of axial images. Images of central airways were printed on film using appropriate algorithms. The number of axial slices collected from patients ranged between 200 and 250. Examination of these images revealed the exact dimensions of the airway lumen, the type of obstruction, obstruction length, airway wall thickness and structures adjacent to the airway. Examination of these images defined size and type of the stent (Figure 1A, Figure 1B).

**Figure 2:**
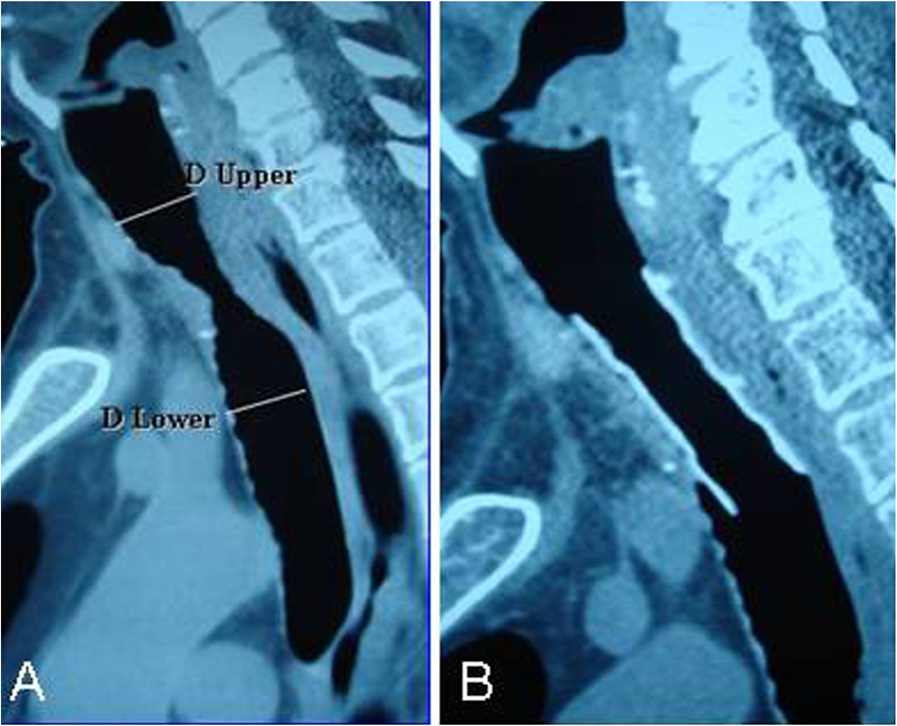
**A MDCT taken on arrival with complaints of shortness of breath identified post-intubation stenosis.** B MDCT image of patient implanted with stenotic silicon stent in the trachea. A 49-year-old male patient had a history of intensive care admission and entubation. MDCT was taken on arrival with complaints of shortness of breath identified post-intubation stenosis. **B** MDCT image of patient implanted with stenotic silicon stent in the trachea.

**Figure 3:**
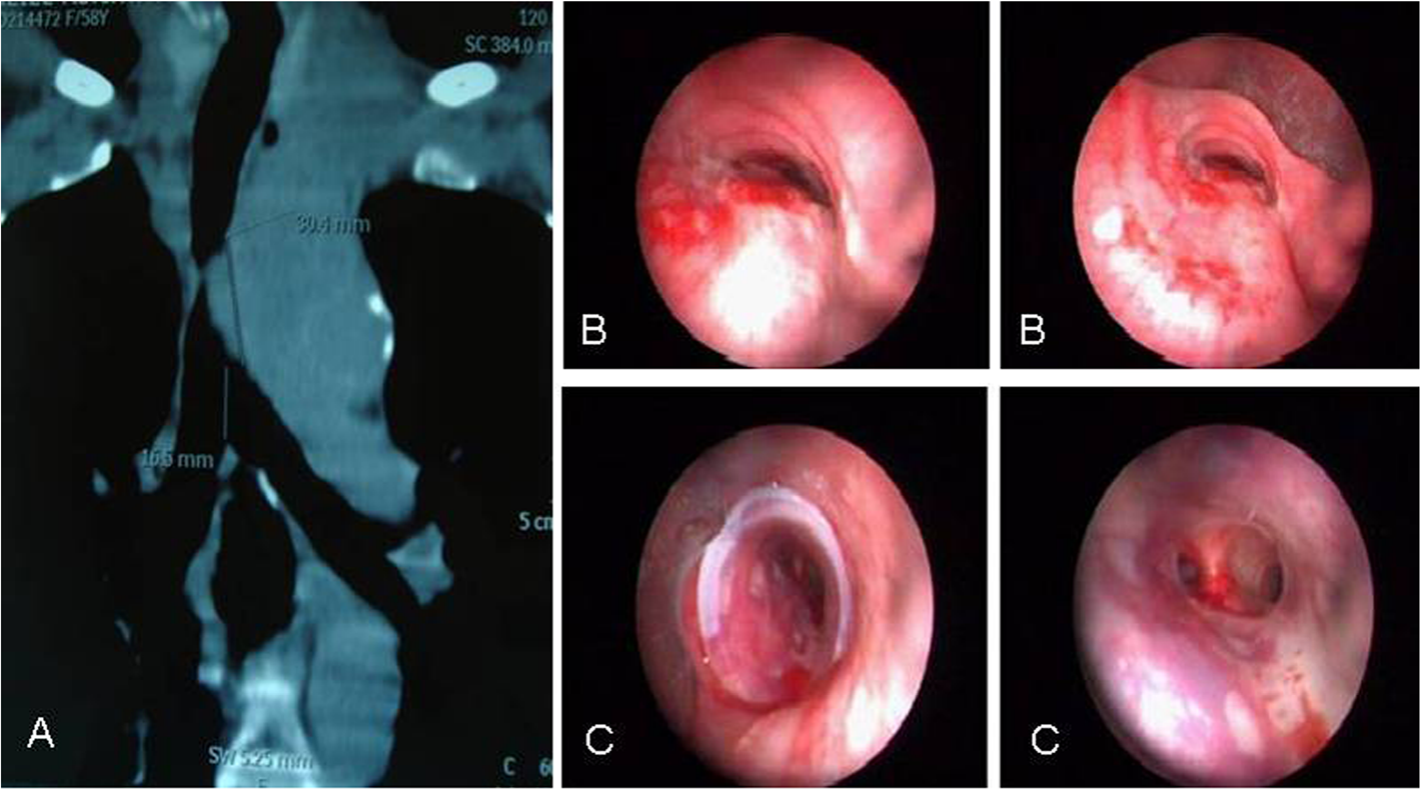
**A MDCT revealed a mediastinal mass compressing the trachea. B** Bronchoscopic images of distal tracheal lumen obstructed nearly 95% due to extrinsic compression. **C** Bronchoscopic images after placing Dumon silicon stent in the trachea. A 59-year-old female patient had a history of surgery for esophageal cancer. MDCT revealed a mediastinal mass compressing the trachea. Prior to endobronchial treatment she was transferred to an intensive care unit after orotracheal intubation because of respiratory failure. **B** Bronchoscopic images of distal tracheal lumen obstructed nearly 95% due to extrinsic compression. **C** Bronchoscopic images after placing Dumon silicon stent in the trachea. In Figure 3C it has shown as the patient's condition improved dramatically after the treatment of endobronchial stenosis. The patient was discharged from the ICU.

Healthy airways proximal and distal of the tracheal obstruction were evaluated with the help of coronal and sagittal reconstructed MDCT images. Obstruction length was measured using these reconstructed images and the stent length was determined by adding 10 mm to the proximal and to the distal tips of the obstruction for tracheal stents and 5 mm for the bronchial stents. The diameters of the airways 10 mm proximal and 10 mm distal to the obstruction were measured from coronal or sagittal reconstructed images as D upper and D lower and the stent diameter (D) was determined from the average between D upper and D lower (Figure 2A, Figure 2B, Figure 3A). Considering the stent length and location helped us to identify a suitable stent size and an optimal position.

## Results

Endobronchial stenosis in our patients were located as follows: 29 (51.8%) tracheal, 13 (23.2%) tracheobronchial and 14 (%25) bronchial (Table 2). Endobronchial stenosis were intrinsic in 23 patients (41.1%), extrinsic in 22 patients (39.3%), and mixed in 11 patients (19.6%) (Figure 3B).

Stents were implanted in all patients undergoing endobronchial treatment (Figure 3C). MDCT helped to determine stent size, length and type. Stent migration as a complication was identified in one case that subsequently underwent stent revision.

## Discussion

Although traditional axial CT imaging meets the requirements for evaluating most airway diseases, several limitations are related to axial imaging. These include limitations to the detection of understated airway stenoses; underestimation of the craniocaudal extent of lesions and inability to define complex 3D relationships of the airways [5,7]. An exact identification and characterization of diseases of the airways, from the point of view of accurate measurements, is important for both the diagnosis and the potential surgical approach to these diseases. Therefore, the disadvantages of axial images impose important limitations on the evaluation of patients with certain diseases of the airways, particularly airway stenoses and complex airway abnormalities which are the most common diseases in the field of interventional pulmonology [8].

MDCT can help in the quick, non-invasive, efficient evaluation of tracheobronchial airways. The system provides the bronchoscopes a more anatomically meaningful display of complex structures and provides also important information before a procedure in a number of pathological situations, including airway neoplasms and congenital airway anomalies. MDCT also offers non-invasive imaging for post-procedure follow up examinations. The sensitivity of this technique to identify airway anomalies including neoplasms is 97%. MDCT identifies intra and extra-luminal tumor protrusions as well as the presence of post-obstructive complications. Furthermore, it can provide simpler staging of the illness by pointing out lymphadenopathy and distant metastasis, and offer guidance to biopsies [5,8,9].

Virtual bronchoscopy, a combination of MDCT axial and 2 dimensional reformation images, is a helpful imaging method for identifying the ideal biopsy location for endobronchial lesions, especially those that do not result in mucosal or airway distortion [4,8,10].

Most cases presenting with airway obstruction by endoluminal tumor growth can be treated endoscopically using laser, cryotherapy etc. A stent is needed if airway obstruction is remarkably caused by external compression. Tracheobronchial stents have three main indications [11]:

1. Ensuring cleared central airways from external compression or internal obstruction.

2. Supporting deficient cartilage dynamics in tracheobronchomalacia cases.

3. Repairing fistulae and leaks between central airways and the oesophagus or pleural cavity.

MDCT offers important information before and after stent implantation. Airway stents remain in their location through the radial pressure they apply on the airway wall. This can cause bronchial ischemic irritation, granuloma formation, hemoptysis, ruptures or other complications. On the other hand small stent sizes will not apply enough pressure to the airway, presenting the risk of migration. This is why it is very important to select optimal stent diameter, length, location and size [12,13]. Reconstructed images will help to determine whether obstruction is intraluminal or extra luminal. MDCT will also help to show whether a patient is suitable for surgery or palliative treatment. It can contribute to decisions about stent size, length and type for patients qualified for endobronchial stents. Size (diameter and length) and type of the stents that are available will differ between healthcare centers. Some centers perform diagnostic bronchoscopy prior to stent implant, however this exposes patients to an additional invasive diagnostic procedure [13,14]. Our study evaluated MDCT images of 56 patients that helped selecting stents of suitable size and shape. MDCT is also just as effective in imaging airways following stent implantation. Besides illustrating the stent location, shape and passage, it helps to define clearly their relation with neighboring airways. MDCT is effective in identifying stent complications like malposition, migration, fractures, incompatible size, severe stent granulation tissue development, on-going stenosis, stent compression or recurrence of local malignity [13]. In our study MDCT identified stent migration and granulation tissue on one patient. Compared to bronchoscopy MDCT is 88% to 100% sensitive and 100% specific in identifying stent complications. Moreover, it has also an essential complementary role to conventional bronchoscopy, providing guidance of bronchoscopic interventions, and offering a noninvasive method for post procedural surveillance [15,16]. The limitation of our study is that it is a retrospective study and does not have a comparison method for the sizing of the stents.

## Conclusion

In conclusion MDCT helps to determine size, length and type of stents for patients suitable to undergo endobronchial stent implants. It is a helpful, non-invasive and reliable imaging method for the evaluation and follow up of the patients before and after endobronchial treatment.

## Competing interests

The authors declare that they have no competing interests.

## Funding

No funding or grant was received.
